# The Regulation between CD4^+^CXCR5^+^ Follicular Helper T (Tfh) Cells and CD19^+^CD24^hi^CD38^hi^ Regulatory B (Breg) Cells in Gastric Cancer

**DOI:** 10.1155/2022/9003902

**Published:** 2022-10-26

**Authors:** Ying Zhang, Jun Wu, Haibo Zhang, Chen Wu

**Affiliations:** ^1^Department of Oncology, The Third Affiliated Hospital of Soochow University, Changzhou, Jiangsu 213003, China; ^2^Department of Oncology, Affiliated Hospital of Jiangnan University, Wuxi, Jiangsu 214000, China; ^3^Department of Ophthalmology, People's Hospital of Ziyang, Ankang, Shanxi 725399, China; ^4^Department of General Internal Medicine, People's Hospital of Ziyang, Ankang, Shanxi 725399, China

## Abstract

**Purpose:**

T follicular helper (Tfh) cells and regulatory B (Breg) cells are reported to play essential roles in humoral immunity, especially in inflammation, autoimmune diseases, and cancer. Hence, we sought to investigate the involvement of CXCR5^+^CD4^+^ Tfh cells and CD19^+^CD24^hi^CD38^hi^ Breg cells in gastric cancer.

**Methods:**

The blood samples were obtained from 36 gastric cancer patients and 18 healthy individuals. The percentage of Tfh cells (Tfh%) and Breg cells (Breg%) was detected via flow cytometry, while IL-21, IL-10, and CXCL13 levels were examined with ELISA. The association between them and clinical parameters of patients was also assessed. The in vitro Tfh-B cell coculture experiments were performed for six days, and then, Tfh%, Breg%, and cytokines were valued by flow cytometry and ELISA, respectively.

**Results:**

Tfh%, Breg%, and CXCL13 level were significantly increased among gastric cancer patients. Moreover, higher Tfh% was associated with lymphatic metastasis, patients' worse outcomes and Breg%. Tfh differentiation and CXCL13 were upregulated by cocultured B cells in vitro, while Tfh cells seem to not participate in Breg cell differentiation from B cells.

**Conclusion:**

Altogether, increased Tfh and Breg cells could be involved in immune suppression in gastric cancer. Moreover, B cell may be a potential regulator for Tfh differentiation, while Tfh cells had no significant effects on the regulation of Breg cells.

## 1. Introduction

Gastric cancer is reported as one of the most common types of cancer and the fourth leading cause of cancer death [1], with particular high incidence and mortality rates in East Asia, including China [2]. Even though early-stage gastric cancer can be cured with surgical or endoscopic resection, most proportion of gastric cancer patients in China are often first diagnosed at advanced stages, whose prognosis is significantly poor [3]. Immunotherapy has been recognized as a promising strategy to change this situation with the improvement of antitumor immune responses [4]. Therefore, it is important to explore the mechanisms of immunoregulation in gastric cancer, in order to enhance the effectiveness of immunotherapy or develop novel treatment strategies.

T follicular help (Tfh) cells are a subgroup of CD4^+^T cells primarily found in the germinal centers of human tonsils [5], which play a key part in humoral immunity. Naive T cells would differentiate into Tfh cells through upregulation of B cell lymphoma 6 (Bcl-6), after getting antigen presentation from dendritic cells (DCs) [6]. Tfh cells then migrate into B cell follicles in response to the specific chemokine ligand 13 (CXCL13) with the increased expression of chemokine (C-X-C) receptor 5 (CXCR5) [7]. In the germinal center, Tfh could assist activation, proliferation, and differentiation of B cell into plasma cells and thus regulate host antibody response [8]. As reported, CD4^+^CXCR5^+^ T cells in human blood shared the same markers and functions with GC Tfh cells, suggesting that peripheral blood CD4^+^CXCR5^+^ T cells may represent the circulating compartment of Tfh-like cells [9]. In breast cancer, Tfh cells are greatly infiltrated in tumor tissues and correlated with disease-free survival of patients [10]. However, Tfh had been found significantly increased with poorer prognosis among patients in other solid tumors, such as non-small-cell lung cancer (NSCLC) [11], hepatocellular carcinoma (HCC) [12], and B cell lymphoma [13]. As reported, the proportion of Tfh cells in the peripheral blood of gastric cancer patients was higher than those of healthy people, which was also related to lymph node metastasis, low differentiation, and stage III-IV, suggesting that Tfh cells may play an important role in the development of gastric cancer [14].

Usually, B cells are described as the main effector members in humoral immunity, capable of suppressing the progression of tumor through the secretion of immunoglobulins, triggering T cell immune response, and directly destroying malignant cells. However, regulatory B (Breg) cells, as a newly designated group, appear to prevent T cell differentiation and promote tumor growth via transforming growth factor-*β* (TGF-*β*) and/or interleukin-10 (IL-10) release [15]. As known, T cell Ig and mucin domain 1 (Tim-1) could identify IL-10-producing Breg cells, which is also required for optimal production of IL-10 in Breg cells [16, 17]. Researches have reported that Breg cells were implicated in regulating immune responses especially in inflammation, serious infection, autoimmune diseases, and cancer [18–20]. As shown in hepatocellular carcinoma, the accumulation of TIM-1^+^Breg cells in tumors were associated with advanced disease stage and reduced patient survival [21]. Breg cells were also found increased in the esophageal squamous cell carcinoma, with the production of IL-10 [22]. Similarly, Breg cells were elevated in cervical cancer and associated with disease progression and metastasis, which could inhibit the cytotoxicity of CD8^+^ T cells [23]. Moreover, Bregs were highly enriched in tumor tissue and peripheral blood of gastric cancer and could suppress the proliferation of autologous CD4^+^ T cells [24].

Although Tfh and Breg cells have been studied in these diseases, the associations between these cells have not been clear. It has been shown that Tfh cells could secrete IL-21 and thus facilitate Breg cell differentiation and production of IL-10 in systemic lupus erythematosus (SLE) [25]. Inversely, Breg cells could control Tfh cell maturation, expand follicular regulatory T cells, and inhibit the Tfh cell-mediated antibody secretion [26]. Nevertheless, the associations among Tfh and Breg cells in gastric cancer have not been extensively elucidated. We have no idea whether Tfh cells would interact with Breg cells to remodel the immunosuppressive microenvironment in gastric cancer.

Therefore, we aimed to compare gastric cancer patient's circulating Tfh cells and Breg cells with healthy individuals in the present study and investigate their association with different clinical and pathological variables within gastric cancer patients. Moreover, Tfh-B cell coculture experiments were conducted in vitro to explore the regulatory relationship between Tfh cells and Breg cells in order to improve the efficacy of immunotherapy for gastric cancer.

## 2. Materials and Methods

### 2.1. Patients and Controls

There were 36 gastric adenocarcinoma patients in the Third Affiliated Hospital of Soochow University enrolled between November 2020 and August 2021. The pathological diagnosis was made by experienced pathologists. Before undergoing any chemotherapy or immunotherapy, peripheral blood was collected from the patients. The tumor stage of patients was evaluated with tumor-node-metastasis (TNM) staging system from American Joint Cancer Committee. Controls were composed of 18 age and gender matched healthy individuals with no history of autoimmune disease or malignancy. The peripheral venous blood used in this study was obtained after conventional laboratory tests, which was admitted in line with the codes of ethics of the Helsinki Declaration. Informed consent for this study was obtained from all participants. Our research was approved by the Third Affiliated Hospital of Soochow University ethics committee.

### 2.2. Isolation of Peripheral Blood Mononuclear Cells (PBMCs)

Peripheral blood samples from patients and healthy controls were harvested in EDTA tubes. The blood was then layered on the top of a standard lymphocyte separation density reagent (Tbd Science, China, LTS10770125) at 2 : 1 volume ratio and centrifuged at 1500 rpm for 30 min until PBMCs were isolated. PBMCs were resuspended and conserved in RPMI-1640 culture medium (Thermo Fisher Scientific, USA, A4192301) after being washed twice.

### 2.3. Flow Cytometry

Human PBMCs were stained with anti-CD4-APC (BioLegend, USA, 300514), anti-CXCR5-PE/cyanine7 (BioLegend, USA, 356924), anti-CD19-FITC (BioLegend, USA, 392508), anti-CD24-APC (BioLegend, USA, 311118), and anti-CD38-PE/cyanine7 (BioLegend, USA, 356608) at 4°C in dark for 30 min to determinate Tfh cell and Breg cell number. The cells were washed with plain PBS (Hyclone, China, SH30256.01B) and then analyzed with a 6-color FACS Calibur (BD Biosciences, USA), utilizing Flowjo v10 software (Tree Star, USA). Tfh% was classified as the percentage of CXCR5^+^ cells in CD4^+^ lymphocytes, and Breg% was classified as the percentage of CD24^hi^CD38^hi^ cells in CD19^+^ lymphocytes. Moreover, IL-10 level (Lushi, China, 20210446) in medium supernatant was also assessed with antibody-coated microspheres via flow cytometry.

### 2.4. Enzyme-Linked Immunosorbent Assay (ELISA)

Serum and culture supernatant for further researches were gathered and stored at −20°C. Commercial ELISA kit was used to measure the concentrations of CXCL13 (Abcam, USA, ab269370) and IL-21 (Abcam, USA, ab119542) according to the instructions from the manufacturer. The absorbance at 450 nm wavelength was read with the spectrophotometer (Eppendorf, Germany).

### 2.5. Cell Coculture

**Figure 1:**
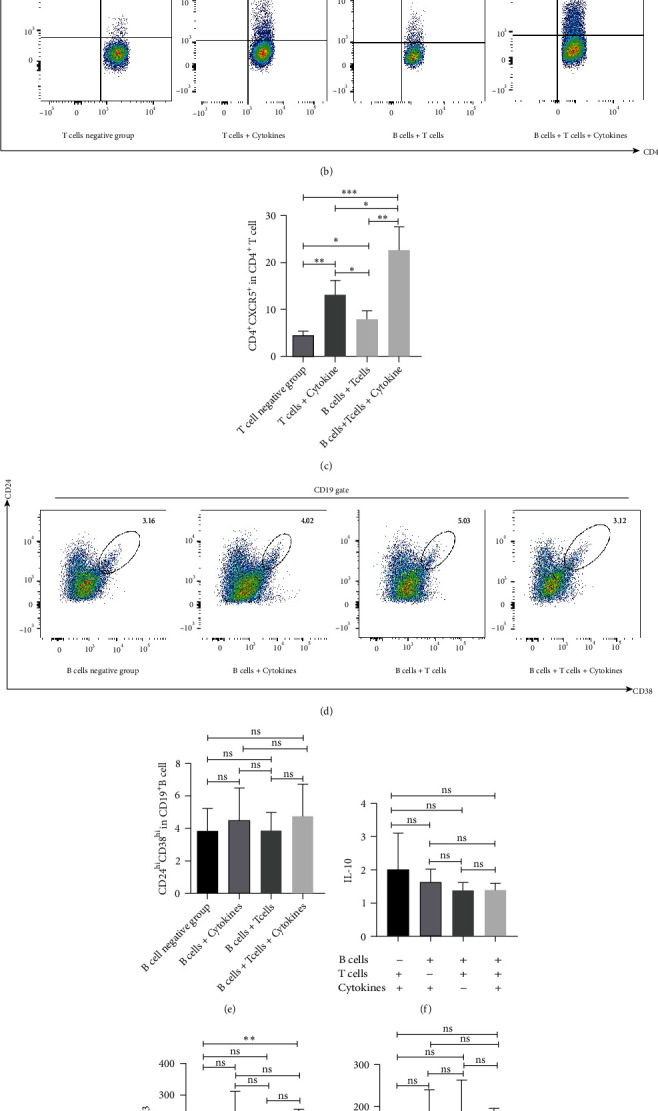
B cell facilitate Tfh cell differentiation and CXCL13 secretion in vitro. Purchased T cells and B cells were obtained from venous blood of healthy people. Tfh cells were stimulated with cytokines and with or without B cell cocultured (a). Tfh cells' gate (b) and its percentage variation in the coculture groups (c). Breg cells' gate (d) and its percentage variation in the coculture groups (e). The level of IL-10 (f), CXCL13 (g), and IL-21 (h) in the coculture groups. Tfh: follicular help T cells; Breg: regulatory B cells; IL-10: interleukin-10; CXCL13: C-X-C motif chemokine ligand 13; IL-21: interleukin-21; ^∗^: <0.05; ^∗∗^: <0.01; ns: no significance.

Fresh primary CD4^+^ T cells and CD19^+^ B cells of normal human were positively selected by magnetic activated cell sorting purchased from OriCells (Oribiotech, China). CD4^+^ T cells and CD19^+^ B cells purity were about 90%. According to the previous studies [27–29], we stimulated T cells into Tfh cells with anti-human CD3 (5 *μ*g/ml, BioLegend, USA, 317326), anti-human CD28 (1 *μ*g/ml, BioLegend, USA, 302933), IL-12 (1 ng/ml, PeproTech, USA, 200-12), IL-6 (10 ng/ml, PeproTech, USA), TGF-*β* (5 ng/ml, PeproTech, USA, 100-21), and IL-23 (10 ng/ml, PeproTech, USA, 200-23) for 6 days (Figure 1(a)). Stimulated T cells were then cocultured with B cells at a ratio of 1 : 1 for 6 days, and the cells were analyzed via flow cytometry as described above, while medium supernatant was collected for ELISA analysis.

### 2.6. Statistical Analysis

SPSS software (version 25.0) was employed for all statistical analyses. Quantitative variables were expressed as median (P25, P75) or as means ± SD. The Mann-Whitney *U* test and one-way ANOVA test were used to figure out statistically significant differences between the two groups. Correlation analysis between Tfh cells and Breg cells was employed by Spearman's correlation analysis. Cutoff Finder [30] is a freely available web application determining cutoff points and thus stratifying patients into two groups (http://molpath.charite.de/cutoff). The prognostic value of Tfh and Breg cells was analyzed via the Kaplan-Meier method. Probability values < 5% have statistical significance.

## 3. Results

### 3.1. Increased Proportion of Tfh and Breg Cells in Gastric Cancer Patients

**Figure 2:**
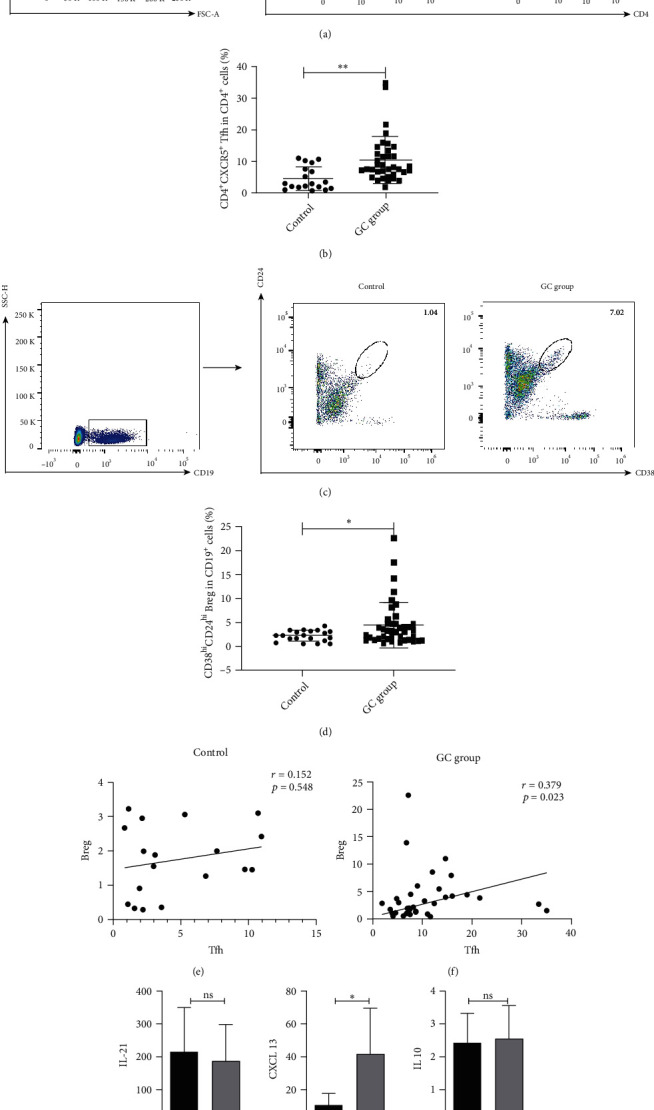
Proportion of Tfh and Breg cells in gastric cancer patients. Blood samples were collected from 36 cases of gastric cancer patients and 18 cases of healthy people. Representative flow cytometric results were shown. Tfh cells were classified as CXCR5^+^CD4^+^ T cells (a), while Breg cells were classified as CD19^+^CD24^hi^CD38^hi^ cells (c). Compared to control individuals, significantly increased percentage of Tfh cells (b) and Breg cells (d) was found in gastric cancer patients (*P* < 0.05). A positive association between Tfh% and Breg% was found in gastric cancer patients (e, *P* < 0.05), rather than in the healthy group (f, *P* > 0.05). Higher CXCL13 level was in the gastric cancer group than in the healthy group (g, *P* < 0.05). IL-21 and IL-10 levels have no significant difference (h and i, *P* > 0.05). Tfh: follicular help T cells; Breg: regulatory B cells; CXCL13: C-X-C motif chemokine ligand 13; IL-21: interleukin-21; IL-10: interleukin-10; ^∗^: <0.05; ^∗∗^: <0.01; ns: no significance.

To investigate the proportion of Tfh and Breg cells in gastric cancer patients and healthy groups, the gating strategies presented in Figures 2(a) and 2(c) were used. We discovered that the proportion of Tfh cell in the gastric cancer group was higher, respectively, than that in the healthy control (Figure 2(b), 7.93% (6.33%, 13.12%) vs. 3.04% (1.85%, 8.18%), *P* = 0.003). This elevation was also found in the percentage of Breg cells compared to the healthy group (Figure 2(d), 2.42% (1.09%, 4.36%) vs. 1.71% (0.79%, 2.74%), *P* = 0.037). Moreover, our results demonstrated that Tfh had correlation with Breg cells in gastric cancer patients (Figure 2(f), *r* = 0.379, *P* = 0.023) rather than in the control group (Figure 2(e), *r* = 0.152, *P* = 0.548). The level of CXCL13 was also increased in gastric cancer patients (Figure 2(h), 44.52 (16.89, 63.26) vs. 8.62 (0.22, 48.25), pg/ml, *P* = 0.017), while there was no difference in the IL-21 and IL-10 levels (Figure 2(g), 171.31 (109.13, 268.31) vs. 200.47 (91.71, 314.74), pg/ml, *P* = 0.515; Figure 2(i), 2.28(2.01, 2.76) vs. 2.09(1.80, 3.08), pg/ml, *P* = 0.295). Our findings indicated that Tfh and Breg subsets along with CXCL13 might participate in the pathogenesis of gastric cancer.

### 3.2. The Relationship between Tfh Cells and Breg Cells and Clinical Features of Gastric Cancer Patients

We next sought to determine whether Tfh, Breg, and Tfh/Breg were associated with clinical characteristics including age, gender, tumor size, metastatic lymph nodes, TNM stage, differentiation, and CEA antigens in gastric cancer patients. As shown in Table 1, the proportion of Tfh was higher in patients with lymphatic metastasis (*P* < 0.05). No significant association was found between Tfh and other studied factors (Table 1, *P* > 0.05). In addition, Breg was not associated with patients' clinical parameters (Table 1, *P* > 0.05).

### 3.3. The Association between Gastric Cancer Patients' Prognosis and Tfh and Breg

**Figure 3:**
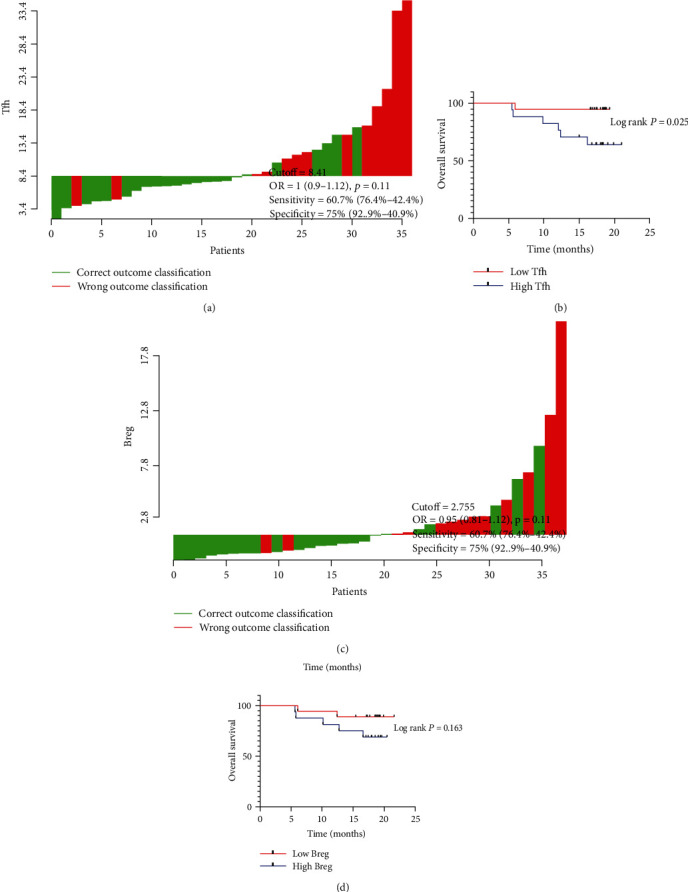
The overall survival (OS) curve for gastric cancer patients according to Tfh and Breg expressions. Cutoff Finder for the optimal cutoff values of Tfh% and Breg%. The cutoff value of Tfh was 8.41% with the sensitivity of 60.7% and the specificity of 75.0% (a), while Breg was 2.755% with the sensitivity of 60.7% and the specificity of 75.0% (c). The patients were stratified into two groups with cutoff values, respectively. The Kaplan-Meier analyses for Tfh and Breg in gastric cancer showing low Tfh% were associated with better prognosis (b, *P* < 0.05), while the percentage of Breg had no significant prognostic value (d, *P* > 0.05). The log-rank test calculated *P* values. Tfh: follicular help T cells; Breg: regulatory B cells.

The optimal cutoff points for Tfh and Breg cells were 8.41% and 2.755%, respectively, according to the Cutoff Finder (Figures 3(a) and 3(c)). Patients then were divided into 2 groups using the cutoff value. Patients with low Tfh proportion showed a significantly better outcome under the Kaplan-Meier analysis (Figure 3(b), *P* = 0.025). There was a better prognostic trend in patients with low Breg proportion, but value has no statistical significance (Figure 3(d), *P* = 0.163).

### 3.4. B Cells Promote Proliferation of Tfh Cells and Secretion of CXCL13 In Vitro

Considering the relationship between Tfh cells and Breg cells in gastric cancer, we evaluated mutual regulatory mechanism between both via human Tfh-B cell coculture in vitro (Figure 1(a)). Tfh cell differentiation from CD4^+^ T cells was successfully induced with stimulation of cytokines mentioned, and the highest rate was almost 13.2% at day 6. Compared with the T-B cell coculture group as well as cytokine-stimulated B cell culture group, the Tfh-B cell coculture group showed no significant increase in proportion of Breg cells (Figures 1(d) and 1(e)). Inversely, in the Tfh-B cell coculture group, there were nearly 26.5% CD4^+^ T cells transformed into Tfh cells (Figures 1(b) and 1(c)) with higher CXCL13 level (Figure 1(g)), while the secretion of IL-10 and IL-21 had no significant changes (Figures 1(f) and 1(h)). Our researches indicated that Tfh cells may not be a major regulator in IL-10-producing Breg cell differentiation from CD19^+^ B cells. On the contrary, B cells may play an important role in Tfh cell differentiation, promoting the secretion of effector molecule CXCL13.

## 4. Discussion

Recently, increasing interest has drawn to the role of Tfh and Bregs in the tumor immune microenvironment. During our study, we investigated the frequency of Tfh and Breg cells in the venous blood of gastric cancer patients. As we expected, Tfh% and Breg% were higher significantly in patients with gastric cancer than in the healthy group. Moreover, increased Tfh% was found relevant to lymphatic metastasis and Breg proportion in gastric cancer. Based on these results, we inferred that Tfh and Breg cells may be possible diagnostic indicators in gastric cancer patients.

Tfh cells appear to be extensively implicated in the pathogenesis and development of multiple malignancies. As known, tumor-promoting functions and malignant B cells rescue of Tfh cells were discovered in hematological malignancies [31], and CXCR5 CAR-T cells targeting Tfh cells improved the therapeutic effect of immunotherapy in B cell non-Hodgkin's lymphoma (B-NHLs) [13]. Previous studies have found increased Tfh frequencies in solid malignancies, including NSCLC [11], HCC [12], and gastric cancer [14], which are consistent with our results. However, unlike the positive prognostic value in breast cancer [10] and colorectal cancer [32], our study found that a lower Tfh% was associated with better patients' overall survival (OS), indicating that Tfh cells may be a negative prognostic mark of gastric cancer patients. The differences between previous studies and ours may result from tumor heterogeneity and ununified types of Tfh cells, which need to be further explored.

Breg cells are known to maintain immune tolerance with the release of IL-10 and other anti-inflammatory mediators [33]. It has been investigated that Breg cells could convert effector T cells into Tregs via TGF-*β*1 [34]. In addition, Breg could strongly suppress CD8^+^ T cell activity with high expressed IL-10 level [21]. Emerging evidence suggested that Breg cells were involved in the development of cancer. Lee-Chang et al. [35] confirmed that tumor-induced Breg cells can induce Foxp3^+^Treg cell proliferation and differentiation in breast cancer and participate in lung metastasis of breast cancer through TGF-*β*-dependent pathway. Qian et al. [36] reported that circulating CD5^+^CD19^+^IL-10^+^ Breg cells was significantly upregulated in esophageal cancer patients and was related to the tumor stage of patients. Similarly, Shao et al. [37] discovered that CD19^+^CD24^+^CD38^+^ Breg cells were increased in HCC, and the increased Breg cells were closely related to the advanced tumor classification and vascular infiltration of the patients. In our study, we also found increased CD19^+^CD24^hi^CD38^hi^ Breg cells in gastric cancer, and there was no significant association with patients' clinical features. Moreover, Breg cell-related IL-10 was not upregulated in gastric patients. As to the prognostic values, there was no meaningful relationship between patients' OS and Breg%, but with slight tendency of difference. The small sample bias and the short follow-up times may explain these differences, and further studies about Breg cells and IL-10 in gastric cancer are needed.

It has been established that tumor progression is the result of interactions between different cell types. As we mentioned before, after migrating into B cell follicles, Tfh cells could aid the growth of B cells within the germinal center. Gu-Trantien et al. [38] reported that Tfh cells may promote local memory B cell differentiation and convert immune suppression to adaptive antitumor humoral responses in the breast cancer microenvironment. Yang et al. [25] discovered that Tfh cells could derive IL-21 to promote the release of IL-10 and Breg cell differentiation in systemic lupus erythematosus (SLE). Qiu et al. [11] revealed that Tfh cells enhanced CD19^+^ B cells releasing TGF-*α* and IL-10 in NSCLC patients, resulting in further immunosuppression and tumor progression. Conversely, in our in vitro experiment, Tfh cells did not make effects on Breg differentiation from B cells and not promote the production of IL-10, suggesting that Breg cells may not be the downstream cells of Tfh cells. As mentioned, Tim-1 is required for optimal production of IL-10 in Breg cells; further Tim-1 blockade experiments would explain more about the activity of Bregs and IL-10 in coculture experiment.

However, we found that there were tightly regulated bidirectional interactions between Tfh cells and B cells. Interestingly, our results revealed that Tfh differentiation and secretion of CXCL13 were significantly promoted by B cells in the coculture group. Previous studies showed that pre-Tfh cells would transform into Tfh cells at the interfollicular zone after a second stimulation by B cells following dendritic cells (DCs) presenting antigen to native CD4^+^T cells [39, 40]. In this process, B cells that express low affinity BCRs could interact with pre-Tfh cells and induce Tfh differentiation with stronger ICOS-ICOSL channel [41]. Sheng et al. [42] reported that virus-like particle-modified B cells were the dominant antigen-presenting cells within naive CD4^+^ T cell activation, which were sufficient to induce Tfh cell development in the absence of DCs. Arroyo and Pepper [43] also discovered that B cells were both sufficient and necessary for Tfh-dominant response generation during Plasmodium infection. Based on these results, we hypothesize that B cells may promote Tfh cell differentiation and mature and inhibit functions of effector T cells in return, which would contribute to immunosuppressive environment in gastric cancer.

There are few limitations to this study. Firstly, only 36 patients were enrolled, and follow-up time was short. Thus, our further research will enroll more patients and prolong the follow-up time of patients to better support our findings. Secondly, the phenotype of Breg cells has not been unified; we only tested CD19^+^CD24^hi^CD38^hi^ Breg cells instead of other phenotypes. Moreover, due to technical limitations, Tfh cells could not be isolated directly, which would reduce the credibility of our results at some extent. Finally, Tfh-B cell interaction was explored in vitro, rather than proven in animal models. In the follow-up study, we will construct B cell-deficient gastric cancer mice models to figure out the underlying regulation mechanisms between Tfh cells and B cells.

## 5. Conclusions

In conclusion, Tfh cells might play a crucial part in suppressing immune response of gastric cancer, while B cells may be the possible regulator for Tfh cell differentiation and improvement for the therapeutic effectiveness.

## Figures and Tables

**Table 1:** Association between Tfh, Breg and clinical characteristics of gastric cancer patients.

Clinical characteristic	*N* = 36	Tfh			Breg		
		M (P25, P75)	*U*	*P*	M (P25, P75)	*U*	*P*
Age (year)			111.0	0.163		136.5	0.570
≤60	14	9.97 (7.08, 15.85)			3.32 (1.02, 4.69)		
>60	22	7.93 (4.77, 11.17)			2.02 (1.14, 3.89)		
Gender			92.0	0.447		84.5	0.295
Male	28	7.93 (6.80, 14.00)			2.77 (1.33, 4.36)		
Female	8	7.77 (4.22, 13.12)			1.20 (1.01, 4.79)		
Lymph node metastasis			22.0	0.035^∗^		37.5	0.182
Negative	32	8.41 (6.83, 14.33)			2.76 (1.19, 4.46)		
Positive	4	4.04 (2.293, 9.41)			1.38 (0.94, 2.56)		
Depth of invasion			48.0	0.421		62.5	0.940
T1-T2	4	5.72 (4.74, 6.77)			1.47 (0.67, 2.70)		
T3-T4	32	8.67 (6.83, 14.33)			2.76 (1.19, 4.46)		
Degree of differentiation			119.0	0.525		124.0	0.643
Poor	25	8.63 (6.48, 14.01)			2.81 (1.10, 4.96)		
Well	11	7.35 (5.26, 11.03)			2.13 (1.06, 2.97)		
Tumor size			107.0	0.127		104.0	0.105
≤5 cm	14	6.84 (4.45, 15.00)			1.82 (1.02, 3.22)		
>5 cm	22	8.87 (7.30, 12.63)			3.05 (1.32, 6.48)		
TNM stage			53.0	0.053		71.5	0.230
I–II	7	5.26 (3.53, 11.03)			1.73 (0.91, 2.97)		
III–IV	29	8.63 (6.91, 14.01)			2.70 (1.22, 4.96)		
CEA (0-5 ng/ml)			84.0	0.287		90.0	0.402
≤5	28	8.67 (6.79, 14.33)			2.91 (1.09, 5.21)		
>5	8	6.91 (4.63, 11.33)			2.02 (1.21, 2.78)		

Mann-Whitney *U* test analysis; significant *P* values are shown with ^∗^. Tfh: follicular help T cells; Breg: regulatory B cells; TNM: tumor-node-metastasis.

## Data Availability

The supporting data of this study are available from the corresponding author on reasonable request.
